# Surgical Sealant with Integrated Shape‐Morphing Dual Modality Ultrasound and Computed Tomography Sensors for Gastric Leak Detection

**DOI:** 10.1002/advs.202301207

**Published:** 2023-06-05

**Authors:** Benjamin Suter, Alexandre H. C. Anthis, Anna‐Katharina Zehnder, Victor Mergen, Jachym Rosendorf, Lukas R. H. Gerken, Andrea A. Schlegel, Eva Korcakova, Vaclav Liska, Inge K. Herrmann

**Affiliations:** ^1^ Nanoparticle Systems Engineering Laboratory Institute of Energy and Process Engineering (IEPE) Department of Mechanical and Process Engineering (D‐MAVT) ETH Zurich Sonneggstrasse 3 Zürich 8092 Switzerland; ^2^ Particles‐Biology Interactions Department of Materials Meet Life Swiss Federal Laboratories for Materials Science and Technology (Empa) Lerchenfeldstrasse 5 St. Gallen 9014 Switzerland; ^3^ Diagnostic and Interventional Radiology University Hospital Zurich University of Zurich Rämistrasse 100 Zürich 8091 Switzerland; ^4^ Department of Surgery Faculty of Medicine in Pilsen Charles University Alej Svobody 923/80 Pilsen 32300 Czech Republic; ^5^ Biomedical Center Faculty of Medicine in Pilsen Charles University Alej Svobody 1655/76 Pilsen 32300 Czech Republic; ^6^ Department of Surgery and Transplantation Swiss HPB Centre University Hospital Zurich Rämistrasse 100 Zurich 8091 Switzerland; ^7^ Fondazione IRCCS Ca' Granda Ospedale Maggiore Policlinico Centre of Preclinical Research Milan 20122 Italy; ^8^ Transplantation Center, Digestive Disease and Surgery Institute and Department of Immunity and Inflammation, Lerner Research Institute Cleveland Clinic 9620 Carnegie Ave Cleveland OH 44106 United States; ^9^ Department of Imaging Methods Faculty of Medicine in Pilsen, Charles University Alej Svobody 80 Pilsen 30460 Czech Republic

**Keywords:** adhesives, anastomotic leak, monitoring, postoperative complications, sensing

## Abstract

Postoperative anastomotic leaks are the most feared complications after gastric surgery. For diagnostics clinicians mostly rely on clinical symptoms such as fever and tachycardia, often developing as a result of an already fully developed, i.e., symptomatic, surgical leak. A gastric fluid responsive, dual modality, electronic‐free, leak sensor system integrable into surgical adhesive suture support materials is introduced. Leak sensors contain high atomic number carbonates embedded in a polyacrylamide matrix, that upon exposure to gastric fluid convert into gaseous carbon dioxide (CO_2_). CO_2_ bubbles remain entrapped in the hydrogel matrix, leading to a distinctly increased echogenic contrast detectable by a low‐cost and portable ultrasound transducer, while the dissolution of the carbonate species and the resulting diffusion of the cation produces a markedly reduced contrast in computed tomography imaging. The sensing elements can be patterned into a variety of characteristic shapes and can be combined with nonreactive tantalum oxide reference elements, allowing the design of shape‐morphing sensing elements visible to the naked eye as well as artificial intelligence‐assisted automated detection. In summary, shape‐morphing dual modality sensors for the early and robust detection of postoperative complications at deep tissue sites, opening new routes for postoperative patient surveillance using existing hospital infrastructure is reported.

## Graphical abstract

Gastric leaks are a greatly feared postsurgical complication. Here dual modality ultrasound and computed tomography sensors for early gastric leak detection is presented. The electronic‐free sensors are fully integrable into adhesive surgical sealant patches as well as patternable, achieving shape morphing contrast changes under conditions of leak, therefore allowing for facile leak diagnostics.

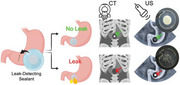

## 1 Introduction

Anastomotic leaks represent the most feared postoperative complications in abdominal surgery.^[^ ^1^, ^2^ ^]^ Leak incidence rates vary significantly, as they depend on multiple factors, including surgeon experience and skills, anatomic location, as well as overall patient condition.^[^ ^3^ ^]^ In the case of gastric surgery, anastomotic leak rates can reach up to 20% for esophagectomies^[^ ^2^, ^4^, ^5^ ^]^ and up to 5% for gastrectomies and bariatric surgeries.^[^ ^6^, ^7^, ^8^ ^]^ Although the chance of developing an anastomotic leak after a gastrectomy or a bariatric surgery is relatively low for an individual patient, the rising numbers of gastric cancer and obesity‐affected patients will likely result in a surge of patients suffering from gastric leaks. A recent worldwide survey estimates that 500 million adults can be categorized as obese (BMI ≥ 30 kg m^−2^).^[^ ^9^ ^]^ While the US alone currently has 30 million adults (9% of U.S. adults aged 20 and above) with extreme obesity (BMI ≥ 40 kg m^−2^),^[^ ^10^ ^]^ these numbers are projected to rise to 10% of the overall US population by 2030.^[^ ^11^ ^]^ For the case of gastric cancer, annually 1.1 million new patients are diagnosed, a number that is predicted to increase to 1.8 million annually by 2040.^[^ ^12^ ^]^ Maintaining that esophagectomy is the most effective treatment for patients with noninvasive esophageal cancer,^[^ ^13^ ^]^ that gastrectomy is the principle treatment for gastric cancer^[^ ^14^, ^15^, ^16^ ^]^ and that bariatric surgery is the only reliable long‐term treatment for extreme obesity,^[^ ^17^, ^18^, ^19^, ^20^ ^]^ it is reasonable to conclude that gastric leaks affect a large number of patients, and will continue to do so.

A variety of suture support^[^ ^21^, ^22^, ^23^, ^24^, ^25^ ^]^ and replacement^[^ ^26^, ^27^, ^28^ ^]^ materials have been developed and employed to support anastomotic sites. However, most of these materials, including fibrin‐based adhesives, have initially been developed for applications to skin wounds or vascular surgery applications,^[^ ^29^, ^30^ ^]^ and are therefore typically prone to digestion by gastrointestinal fluids.^[^ ^31^, ^32^, ^33^ ^]^ Exacerbating the issue, anastomotic leaks are currently only detectable once the patient develops clinical symptoms, such as pain, fever, and overall deterioration of their health condition.^[^ ^34^, ^35^ ^]^ In these cases, the leak typically is already fully developed, and gastric juice has entered the peritoneal cavity of the patient, making the treatment of these leaks even more precarious. First experimental attempts on anastomotic leak detection sensors integrated into sutures rely on complicated electronics and measurement setups,^[^ ^36^, ^37^ ^]^ further complicating clinical translation.

Most recently, a novel adhesion technology based on the formation of tissue‐penetrating polymer networks for the anchoring of hydrogel sealants has been introduced.^[^ ^32^ ^]^ The formation of mutually interpenetrating networks (mIPNs) traversing both the hydrogel sealant patch as well as biological tissue enables unprecedented tissue anchoring even in chemically harsh conditions, including the ones encountered in the stomach.^[^ ^32^, ^38^ ^]^ While this approach offers a promising route to tissue sealing and suture support, it also provides a platform for the incorporation of sensing elements for early leak detection. Previously, such an approach was not feasible due to the early failing of the suture support material and the inability to keep a sensor immobilized on tissue during the leak occurrence. In a first proof‐of‐concept study, echogenic sensing elements were integrated into sealant patches.^[^ ^38^ ^]^ Ultrasound‐based monitoring offers promising prospects, including the possibility for frequent monitoring (owing to the absence of ionizing radiation, low cost, and wide‐spread availability) or even continuous^[^ ^39^ ^]^ monitoring. However, the diagnostic uncertainty in ultrasound imaging, due to the innate 2D nature of ultrasound,^[^ ^40^ ^]^ and the limited imaging depth of ultrasound in obese patients,^[^ ^41^ ^]^ is potentially limiting the diagnostic potential of monomodal echogenic sensors at large. Currently, X‐ray computed tomography (CT) imaging remains the gold standard imaging modality for the assessment of gastrointestinal complications; however, it has significant limitations in the identification of anastomotic site intactness,^[^ ^42^ ^]^ as well as a lack of consensus on radiographic findings associated with leakage^[^ ^43^ ^]^ and exposes the patient to harmful ionizing radiation. Therefore, strategies enabling the unambiguous early identification of anastomotic wound site complications based on designated electronic‐free sensors compatible with standard clinical imaging are urgently needed.

**Figure 1:**
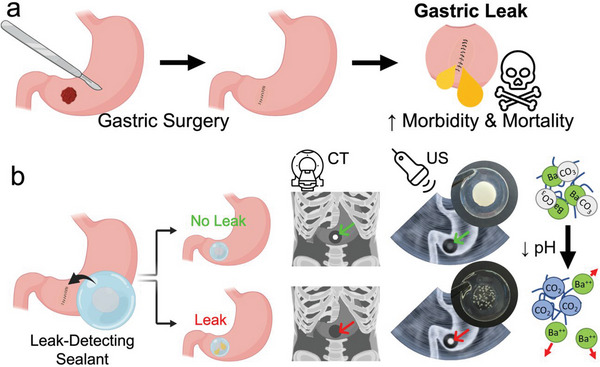
a) Gastric surgeries put the patient at risk for postoperative complications caused by leaking tissue reconnections releasing gastric juice into the abdominal cavity. b) The dual modality gastric leak‐detecting smart sealant allows unambiguous early leak detection via the two clinically most favorable imaging modalities, Computed Tomography (CT) and ultrasound (US). The gastric leak is detected based on contrast changes due to dissolution of barium (or lanthanum) carbonate (BaCO_3_), resulting in a pronounced decrease in CT contrast and a concurrent increase in ultrasound contrast due to the formation of carbon dioxide (CO_2_) bubbles entrapped in the hydrogel matrix.

We designed a surgical sealant containing shape‐morphing leak‐detection sensing elements featuring dual modality properties for ultrasound and (confirmatory) CT based imaging (**Figure** **1**). Barium or lanthanum carbonate sensing elements were integrated into a layered adhesive hydrogel sealant with a nonadhesive backing. Upon contact with the acidic gastric fluid, carbonates decompose and liberate gaseous CO_2_, which remains entrapped in the gel matrix and leads to a distinct increase in ultrasound contrast. Simultaneously, the dissolution of the carbonate leads to dissociation of the high atomic number cation, resulting in a concomitant decrease in X‐ray absorbance and contrast. The sensing elements can be shaped into a diversity of (shape‐morphing) patterns offering high contrast to anatomical structures. Such distinctive sensing element patterns facilitate recognition by the naked eye, and pave the road for (semi‐)automated sensing element recognition by artificial intelligence‐based image analysis algorithms for automated wound site monitoring. The presented sensing elements thus enable reliable and fast leak detection by the two clinically most relevant imaging modalities; the ultrasound imaging modality offers a route to accessible, affordable, close‐meshed, and safe postoperative anastomotic site monitoring capabilities, while the corresponding CT contrast offers validation, enabling imaging with full anatomical context.

## 2 Results

### 2.1 Design of Dual Modality Sensor for Gastric Leak Detection by CT and Ultrasound Imaging

To realize unambiguous and facile leak detection, we designed gastric fluid reactive sensing elements based on hydrogel matrix‐embedded carbonate salts. A selection of carbonate salts was screened for optimal chemical reactivity (no dissolution in peritoneal fluid, full dissolution in gastric juice) and sensor response (maximizing contrast in both CT and ultrasound imaging) (Figure S1, Supporting Information). Low atomic number molecules, such as sodium and calcium ions, exhibited an unfavorable CT contrast response (even at concentrations of up to 30 wt% in the hydrogel matrix), resulting in a low Houndsfield unit (HU) drop post contact with gastric fluid (≤ 45 HU). Furthermore, sodium bicarbonate tends to partially react under nonleak conditions, resulting in the formation of unwanted CO_2_ bubbles entrapped in the hydrogel matrix (Figure S2, Supporting Information). Silver carbonate led to silver chloride precipitation, and therefore also to an unfavorable sensor response (HU values increased by 45 HU when in contact with simulated gastric fluid (SGF) compared to phosphate‐buffered saline (PBS), see Figure S1, Supporting Information). Overall, barium and lanthanum carbonates showed promising changes in contrast in CT and ultrasound signals for the concentration range between 5 and 10 wt%. This concentration range yields relevant changes in the imaging contrast for both modalities,^[^ ^44^, ^45^ ^]^ while still minimizing the weight percentage of carbonate salt loading (Figure S3, Supporting Information). Notably, lanthanum carbonate produces slightly more CO_2_ per mole in comparison to barium carbonate (La_2_(CO_3_)_3_ + 6 HCl → 2 LaCl_3_ + 3 CO_2_ + 3 H_2_O (resulting in 5.5 × 10^−3^ mol of CO_2_ per g of La_2_(CO_3_)_3_) vs BaCO_3_ + 2 HCl → BaCl_2_ + CO_2_ + H_2_O (5.05 × 10^−3^ mol of CO_2_ per gram of BaCO_3_). However, the sensing element response was not only dependent on the type of carbonate salt used, but also on the hydrogel matrix containing the carbonate material. By engineering the embedding matrix, it is possible to control the size distribution of formed CO_2_ bubbles, as well as the homogeneity of the bubble distribution within the embedding matrix. The key parameters determining tissue discernability of the activated sensing elements under ultrasound imaging are the bubble size and count, as well as bubbles being homogeneously distributed throughout the hydrogel. Hence, a large number of small bubbles homogeneously distributed in the hydrogel is easily distinguishable from the tissue, whereas having fewer large bubbles in the hydrogel is not as favorable (Figure S4, Supporting Information). By choosing 20 wt% polyacrylamide as the embedding matrix, the desired CO_2_ bubble parameters were achieved. Additionally, and most importantly, the bubble entrapment and long‐term stability of the bubbles inside the hydrogel were highly dependent on the architecture of the sensing elements. By semiencapsulating the sensing element hydrogel within a secondary 20 wt% polyacrylamide hydrogel layer, CO_2_ bubble retention and ultrasound signal stability were drastically improved to last ≥ 24 h (Figure S4, Supporting Information). In comparison, the ultrasound signal of a nonencapsulated sensing element of identical size immediately started to decrease after reaching a maximal peak at the 1 h time point of SGF incubation and further dropping below its unreacted ultrasound contrast after 24 h of SGF incubation (Figure S5, Supporting Information). In addition to the sensing element composition, the volume of the sensing element was optimized for maximal visibility in ultrasound imaging and under minimal total carbonate salt load of the sensor (Figure S6, Supporting Information). For a cylinder of 4 mm diameter, a 60 µL sensing element had a sufficiently large volume to be efficiently identifiable under ultrasound, regardless of the view plane.

### 2.2 Dual Modality Sensing and Sensor Response Kinetics

**Figure 2:**
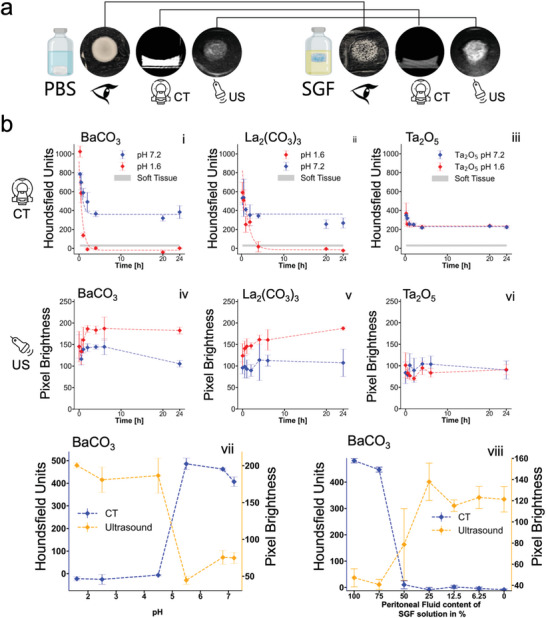
a) Representative examples of nonreacted (left) and fully reacted (right) 60 µL polyacrylamide (PAAm) hydrogels containing 10 wt% barium carbonate (BaCO_3_) after 24 h exposure to phosphate‐buffered saline (PBS) or simulated gastric fluid (SGF), respectively. The resulting changes in contrast after contact with SGF are identifiable by the naked eye, as well as CT and ultrasound. Representative images from *N* = 3 samples shown. b) First row: Time evolution under CT imaging of 60 µL PAAm hydrogels containing 10 wt% BaCO_3_ (i), 10 wt.% lanthanum carbonate (La_2_(CO_3_)_3_) (ii) or 5 wt% tantalum oxide (Ta_2_O_5_) (iii). Second row: Time evolution under ultrasound imaging of identical hydrogel sensing elements (same material order as for CT BaCO_3_ (iv), La_2_(CO_3_)_3_ (v), and Ta_2_O_5_ (vi)). The generated contrast under ultrasound and CT was measured at discrete time points between 0 and 24 h of the hydrogel being exposed to 10 mL of either SGF (pH 1.6) or PBS (pH 7.2). Ta_2_O_5_ was used as a nonreactive control. Third row: (vii) pH sensitivity of the Barium carbonate sensors. 60 µL PAAm hydrogels containing 10 wt% BaCO_3_ were exposed to 10 mL of incubation buffers with varying pH values, ranging from 1.6 (SGF) to 7.2 (PBS). Ultrasound and CT measurements were taken after 24 h of fluid contact. (viii) Specificity of the Barium carbonate sensors. 60 µL PAAm hydrogels containing 10 wt% BaCO_3_ were placed in 10 mL of incubation liquids with varying mixtures of SGF and simulated peritoneal fluid. Ultrasound and CT measurements were acquired after 24 h of fluid contact. *N* = 3 independent experiments performed per condition.

Having established the material makeup and architecture of the dual modality sensor, we fully characterized the sensor response kinetics under simulated leak and no‐leak conditions. Barium carbonate is a promising candidate owing to the fact that barium composites are already established X‐ray contrast agents.^[^ ^46^ ^]^ Lanthanum carbonate has favorable low toxicity^[^ ^47^ ^]^ as well as a higher CO_2_ production capacity per mol when compared to barium carbonate, validating further investigation of the latter. **Figure** **2**b (i, ii, iv, v) shows both barium and lanthanum carbonate sensing elements at 10 wt% concentrations with excellent distinguishability between leak and no‐leak conditions. For ultrasound as well as CT, both barium and lanthanum carbonate showed a signal evolution with excellent stability. Under both imaging modalities, both carbonate sensing elements reached the largest contrast change at the 2 h mark of gastric fluid contact. Thereafter, the signal remained stable for ≥ 24 h. In addition to the two carbonates, tantalum oxide (at 5 wt%) was investigated as a nonreactive reference under ultrasound and CT. Figure 2b(iii, vi) shows that tantalum oxide elements exposed to SGF remain indistinguishable to the tantalum carbonate elements in contact with simulated peritoneal fluid (PBS). This nonreactiveness of tantalum oxide under leak conditions will not only allow for proper signal referencing, but also provide the opportunity to fabricate unique sensing element geometries, capable of morphing into new shapes under leak conditions. This feature significantly improves the diagnostic readout of the sensing elements and will be discussed in more detail below.

### 2.3 Sensitivity and Specificity of Dual Modality Sensor

To assess the feasibility of the designed sensing elements for gastric leak detection, we further characterized the sensor response and investigated the sensitivity and specificity (Figure 2b(vii, viii)). This is crucial for determining the range of conditions under which gastric leaks can be detected. The measurements assessing the dilution of gastric fluid with peritoneal fluid reveal high reaction sensitivity; the sensor is still capable of detecting a gastric leak after gastric fluid is diluted 1: 1 with peritoneal fluid. The excessive dilution of gastric fluid in the envisioned application is an unlikely event, especially because the sensing element remains in close proximity of the gastric leak even during leak conditions, owing to the adhesion enabled by the mutually interpenetrating network that anchors the patch to the defect.

Barium carbonate (and to a slightly lesser extent lanthanum carbonate, Figure S7, Supporting Information) covers a wide range of physiological gastric pH values (Figure 2b(vii)). With the reactivity of the sensing element guaranteed at pH levels at 4.5 or lower, the conditions for the reaction are almost independent of a patients fasting or fed state, as even for elderly patients gastric pH levels can be expected to drop to ≤ 4 within 1 h of a meal.^[^ ^48^ ^]^ Furthermore, this favorable behavior also excludes false positives due to faulty sensor reaction. Our investigations lead us to conclude that both barium (and lanthanum) carbonate sensing elements solely react under pH conditions related to gastric fluid contact.^[^ ^49^, ^50^, ^51^ ^]^ Importantly, these data also illustrate that the two states (leak and no leak) can be efficiently distinguished based on absolute readouts from clinical imaging modalities (i.e., Houndsfield units in CT imaging, where signals change by two orders of magnitude and the pixel brightness in ultrasound). However, taking advantage of the Ta_2_O_5_ reference element, ratiometric imaging may also be used, e.g., to correct for hydrogel swelling. This would remove the potential decrease in signal under CT imaging due to the loss in density related to the initial swelling seen during the early stages (≤ 4 h) of hydrogel incubation of the otherwise highly stable hydrogel (Figure S8, Supporting Information).

### 2.4 Dual Modality Sensing Adhesive Surgical Sealant Patches

The established and characterized sensing element designs were integrated into an open architecture dual layer adhesive patch. The simplistic approach of sensing elements allows significant architectural liberty. To establish the concept of dual modality gastric leak sensing immobilized on the wound site by a surgical sealant, we made use of 20 wt% polyacrylamide (PAAm) to encapsulate the carbonate salt sensors, and for the adhesive support layer in contact with the wound site, replicating the conditions under which the sensing element kinetics were investigated. By using identical materials for the adhesive support layer and the sensing element matrix, foremostly any potential mechanical mismatches between the different patch layers are avoided. Furthermore, PAAm is a promising choice considering the material's biocompatibility.^[^ ^32^, ^52^, ^53^ ^]^

**Figure 3:**
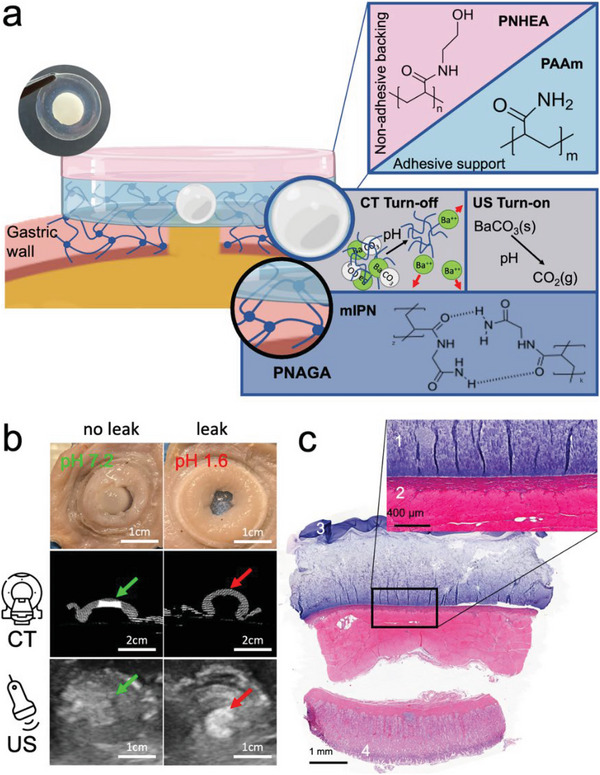
a) Chemical makeup of dual modality sensor patch. Sensors are incorporated directly into the adhesive layer ensuring direct contact with the anastomotic site. The sensing reaction upon contact with gastric fluid is described by the decomposition of the carbonate material, allowing for dissipation of barium ions while concurrently the carbonate ions transform to gaseous CO_2_, which remains entrapped inside the adhesive hydrogel layer, resulting in bubble stabilization. The adhesion of the sensing patch is established by a mutually interpenetrating network. b) Ex vivo tissue application proof of concept. A piece of fresh pig stomach with an 8 mm defect was used as an interface to a 10 mL fluid reservoir filled with PBS (left) and SGF (right), respectively. Both tissue defects were sealed by a dual sensing patch and left overnight. The change in contrast of the sensing element in response to gastric fluid exposure is unambiguously detectable under both imaging modalities. The sensing element contrast decreases under CT and increases under US when in contact with SGF (*N* = 3, data collected from three independent experiments). c) H&E stained porcine stomach biopsy section of the dual modality sensor patch on porcine stomach serosa, with 1 depicting the adhesive hydrogel layer along with the incorporated barium carbonate sensing element, 2 depicting the stomach serosa layer, 3 depicting the nonadhesive backing, and 4 depicting the mucosal layer of the stomach. Representative image from *N* = 3 samples shown.

Unwanted postsurgical adhesion of the surgical sealant patch is avoided by layering a nonadhesive backing layer on top of the adhesive support layer.^[^ ^54^ ^]^ The nonadhesive backing of the patch is made up of 50 wt% poly(*N*‐hydroxylethyl acrylamide) (PNHEA).^[^ ^38^ ^]^ The immobilization of the dual modality leak detecting hydrogel patch onto the wound site is achieved by a mutually interpenetrating network made up of 33 wt% *N*‐acryloyl glycinamide (NAGA) along with the initiator Lithium phenyl‐2,4,6‐trimethylbenzoylphosphinate (LAP), functionable under visible light. Direct wound contact of LAP as well as UV irradiation of LAP in direct contact with wound sites has not shown any adverse implications for wound healing in previous work.^[^ ^55^, ^56^ ^]^ A full display of the complete layered patch architecture along with incorporated dual sensing elements is shown in **Figure** **3**a. As previously demonstrated, the attachment via a mIPN allows for reliable long‐term adhesion under the harshest digestive conditions.^[^ ^32^, ^38^ ^]^ Following standardized T‐peel tests (ASTM standard F 2256‐05)^[^ ^57^ ^]^ the presented hydrogel sealant patch formulation along with mIPN anchorage achieved adhesion of 2.3 ± 0.6 N cm^−1^. Most importantly, the addition of an mIPN only minimally slows the response kinetics of the sensing elements (Figure S5, Supporting Information). This allows full patch sealing and sensing investigations in an ex vivo setting, as shown in Figure 3b. The dual sensing reactivity of a fully assembled surgical sealant patch was investigated by applying a circular hydrogel patch (*d* = 2 cm) along with a centrally incorporated standard cylindrical 10 wt% BaCO_3_ sensing element onto the serosa layer of a porcine stomach tissue sample, covering an 8 mm defect. When brought into contact with SGF from the mucosal layer side of the tissue, the sensing elements reacted completely, yielding a detectable change in the signal of ultrasound and CT. The same signal change did not occur when replacing SGF with PBS. Furthermore, the mIPN‐anchored hydrogel successfully sealed the stomach defect during the entire experiment and firmly attached to the tissue serosa, as confirmed by histological analysis (Figure 3c).

### 2.5 Patterning of Sensors and Imaging in High and Low Dose CT

With the established integrability of the sensing elements into hydrogel sealant patches, we explored possibilities to increase their recognition and detectability when located within a complex anatomically relevant setting. Coupled with the potential of a diagnostic algorithm, sensor patterning would provide further robustness to the detection of gastric leaks. Identifying small amounts of contrast‐generating elements under ultrasound and CT can be difficult. To address this and facilitate the identification of sensing elements under CT and ultrasound imaging, we explored the possibility of assembling sensing elements out of the established reactive and nonreactive elements of BaCO_3_ and Ta_2_O_5_, respectively.

**Figure 4:**
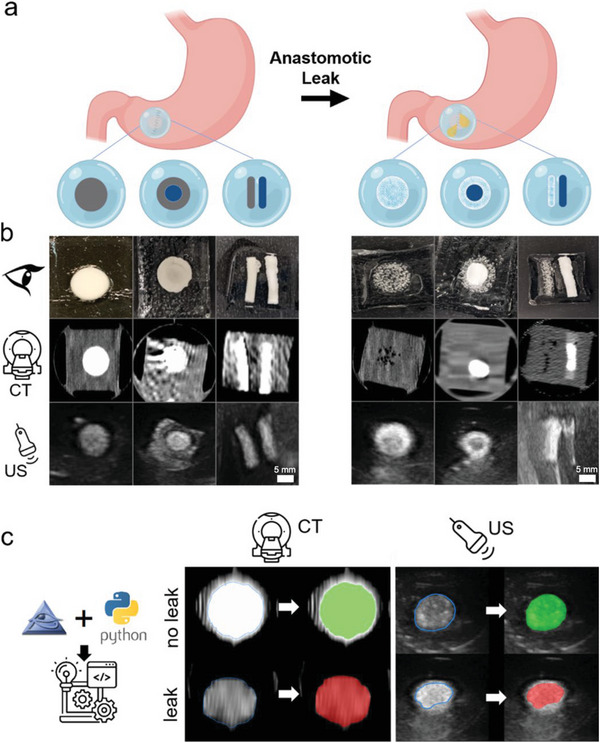
a) (Top) To guarantee easy distinguishability of the dual sensing elements from other anatomical appearances within the human abdomen, reactive geometries are of great benefit. A nonexhaustive list of potential dual sensing element patterns is displayed, made up of reactive and nonreactive components. b) In vitro realization of the above proposed dual sensing element patterns. A nonreacted version (left) is compared to the reacted version (right) visually (top), under computed tomography (CT) (middle), and under ultrasound (US) (bottom). c) Generated CT and ultrasound signal can easily be algorithmically evaluated. By making use of open‐source software, a leak detecting application, capable of classifying the state of the dual sensing element under ultrasound and CT was developed. The application classifies the absolute contrast value of a user selected image region into a leak (red) or no leak (green) category. Representative images from *N* = 3 samples shown.

As shown in **Figure** **4**a,b, the sensing element can not only assume different shapes (circle or line) but its contrast can also morph into a different geometry upon reaction under leak conditions (solid circle to ring element under CT or ultrasound). The combination of the freedom of choice of the sensing element geometry as well as in the change of contrast geometry under leak conditions provides vast opportunities to fine‐tune sensing elements corresponding to the preference of a radiologist. This contributes greatly to optimal detectability, both in terms of discerning the sensing element from human anatomical features, as well as being a reliable detection of gastric leaks.

By leveraging the large change in the absolute signal value (Figure 2), it was possible to create a proof of principle diagnostic software application that classifies the sensing elements into a leak or no leak state. Figure 4c shows the automated color coding of the sensing elements depending on the intensity. In a medical diagnostic image, the sensing element can be outlined, and the software automatically color codes the outlined region with green in the case of no leak and red in the case where the sensing element indicates a leak. This is a crucial step to minimize the possibility of false sensor readouts, leading to undetected leaks, while at the same time paving the way for unassisted leak identification, following previous advancements in artificial intelligence assisted CT and ultrasound data interpretation.^[^ ^58^, ^59^, ^60^ ^]^

### 2.6 Cytocompatibility of Released Ions and CT Dose Risk

**Figure 5:**
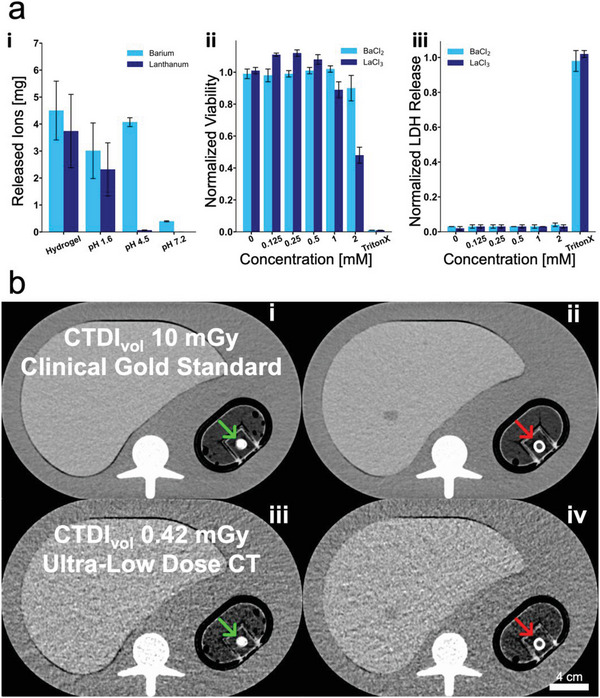
a)(i) Total amount of ions released after hydrogel digestion and ion release as a function of pH for barium and lanthanum carbonate. (ii) Normal human fibroblast cell viability and (iii) membrane integrity as a function of barium (Ba) and lanthanum (La) concentration in the cell culture medium. *N* = 3 independent measurements per condition. b) Comparison of abdominal computed tomography (CT) images acquired with a reference radiation dose level (top) and acquired with ultralow radiation dose (bottom), denoted by their respective volume CT dose index (CTDI_vol_). Sensing elements with a 10 wt% barium carbonate (Ba_2_CO_3_) core and a 5 wt% tantalum oxide (Ta_2_O_5_) outer ring were incubated in phosphate‐buffered saline (PBS) (nonreacted) and simulated gastric fluid (SGF) (reacted) for 24 h, before being placed into a CT phantom simulating adipose tissue. Identical nonreacted (left) and reacted (right) sensing elements were imaged with the two aforementioned CT protocols. Representative image from *N* = 3 samples shown.

Prior to providing in vivo proof of concept of the dual modality sensing patch, we assessed the potential patch cytotoxicity, particularly regarding its released constituents (barium and lanthanum ions). We investigated the cell viability as well as lactate dehydrogenase (LDH) release in human fibroblast cells when exposed to cell medium spiked with the expected release constituents of the dual sensing elements in the form of BaCl_2_. In parallel, we studied the ion release of the carbonate salt dual sensing elements after being immersed in fluids of different pH (1.6–7.2). The theoretical barium ion content of a 60 µL hydrogel containing 10 wt% BaCO_3_ assuming full dissolution is 4.2 mg. As shown in **Figure** **5**a(i), ICP OES of 60 µL hydrogels fabricated from a bulk solution of 10 wt% BaCO_3_ yield a higher mean Ba ion content of 4.5 ± 1.0 mg. This is due to sedimentation of the carbonate salts within the hydrogel precursor prior to polymerization. To guarantee constant initial contrast as well as contrast change, the sedimentation of carbonate salts within the initial hydrogel monomer mix must be considered.

However, by assessing cell viability and observing LDH results from Figure 5a(ii,iii), we identify a normalized metabolic activity of ≥ 0.04 ± 0.01 as well as a normalized toxicity of ≤ 0.9 ± 0.08 for BaCl_2_ up to a concentration of 2 mm. By relating this to the expected ion loading of a 60 µL sensing element containing 10 wt% of BaCO_3_ and considering that even during a fasting state, the human stomach contains on average 30 mL of gastric fluid,^[^ ^61^ ^]^ a 60 µL hydrogel sensing element with a BaCO_3_ loading of almost up to 20 wt% would still convey a nontoxic condition according to our investigation. Furthermore, the United States Environmental Protection Agency (EPA) determined the reference dose for oral exposure (RfD) to Barium to be 2 × 10^−1^ mg kg^−1^ day^−1^, which corresponds to 14 mg day^−1^ for a 70 kg human.^[^ ^62^ ^]^ Therefore, we categorize the preferred 60 µL at 10 wt% BaCO_3_ dual sensing elements as being well below the threshold of cytotoxicity.

To minimize the patient burden and capitalize on the benefits enabled by these dual modality sensors, we evaluated the leak sensing performance under an ultralow radiation dose CT. A volume CT dose index (CTDI_vol_) ≥10 mGy CT scan marks the clinical gold standard for abdominal radiography.^[^ ^63^ ^]^ To mimic the X‐ray attenuation of a human patient, an anthropomorphic abdominal CT phantom^[^ ^64^ ^]^ was modified by replacing the spleen insert with a custom‐built sample holder filled with animal fat, simulating adipose tissue.^[^ ^65^ ^]^ Figure 5b compares standard clinical radiation dose CT images of a nonreacted and a reacted Ta_2_O_5_‐Ba_2_CO_3_ ring shape sensing element (i and ii, respectively) to an ultralow radiation dose CT image of the same sensing elements (iii and iv). At ultralow radiation dose (CTDI_vol_ 0.42 mGy) CT images still show excellent discernability between reacted and nonreacted sensing elements. Following P.C. Shrimpton's published dose length product (DLP) to effective dose (E) conversion coefficient for abdominal CT scans,^[^ ^66^, ^67^ ^]^ the ultralow dose CT would correspond to an E of 0.126 mSv, comparable to 80 days of natural background radiation.^[^ ^68^ ^]^ Considering the gold standard of abdominal CT scans as a radiation exposure benchmark, the ultralow dose CT compatibility of the presented dual modality gastric leak sensors would allow for more than 21 ultralow dose CT scans before reaching the same effective radiation dose of one single CTDI_VOL_ 10 mGy CT scan. This tentatively suggests a theoretical limit of daily CT surveillance of the first 3 weeks postsurgery, which covers the crucial postsurgical period, considering the definition provided by Csendes et al., stating that even late leaks occur already after ≥10 days postsurgery.^[^ ^69^, ^70^ ^]^ The higher CT examination frequency potential along with continuous ultrasound monitoring ability offered by the designed patches underlines the feasibility of drastically reducing the detection time for postsurgical gastric leaks.

### 2.7 In Vivo Proof‐of‐Concept

**Figure 6:**
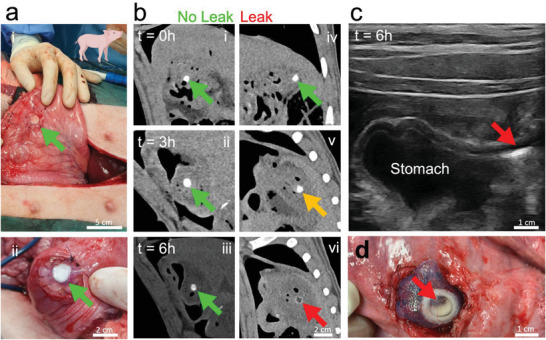
a) Immobilization of dual sensing patches on the stomach serosa layer (i) and on the mucosal layer of the stomach (ii) in a living piglet. To simulate an anastomotic leak condition, circular defects of 5 mm diameter were cut into the mucosal layer providing contact between the dual sensing patch and gastric fluid. b) Computed tomography (CT) image of the control patch with no defect (left) and the patch with a defect (right) at discrete time intervals of *t* = 0, 3 and 6 h (i–vi). Changes in the sensing element shape and contrast occurs after 3 h (v, orange arrow). After 6 h (vi) the dual sensing patch under leak condition shows clear contrast changes by having fully transformed from a circular to a ring shape. c) Ultrasound image of the dual sensing patch under leak condition. The encapsulated carbon dioxide (CO_2_) bubbles yield a bright contrast under ultrasound, rendering the reacted dual sensing patch clearly distinguishable from the surrounding tissue. d) The patch exposed to leak conditions shows bubbles in the center of the circular shape (red arrow). Representative images from *N* = 2 piglets shown.

The functionality of the dual modality leak sensors was demonstrated in vivo in a piglet model. The detectability of dual modality sensors was evaluated along with the sensor capacity to react under leak conditions in a live animal (**Figure** **6**a). Stomach defects were created, and sensing patches were applied to defective and intact stomach tissue sites. CT images were acquired at distinct time points of 0, 3, and 6 h postsurgical implantation of the dual sensing patches. Already after 3 h postsurgery, changes in the shape of the sensing element were visible in the gastric leak scenario.

After 6 h postsurgery, a clear distinction between the unreacted and reacted sensing element is evident by the naked eye based on the CT data. The CT signal of the dual sensing hydrogel patch not in contact with gastric fluid remained unaltered during the entire period of investigation (Figure 6b, no leak). At the 6 h postsurgery time point, the dual sensing signal was investigated by means of ultrasound. A bright spot was observed, clearly distinguishable from the stomach and other abdominal organ tissues, colocalizing well with the CT‐signal (Figure 6c). Following clinical imaging by CT and ultrasound, the abdomen of the piglet was opened for visual inspection of the sensing elements. While the control sensing patches remained unaltered, those in contact with the gastrointestinal fluid leak had the inner carbonate‐containing circle converted into CO_2_ bubbles that remained entrapped in the hydrogel (Figure 6d). The outer ring containing the Ta_2_O_5_ reference remained unaltered and served as a reference enabling straightforward patch and sensor state identification within the abdominal cavity of the piglet. While in‐depth studies on the long‐term fate of the sensor‐containing hydrogel sealants are needed prior to clinical translation, prolonged adhesion of synthetic hydrogel materials to abdominal tissues necessary for such a sensing platform have previously been demonstrated, for hydrogels adhering to intestinal tissue for ≥ 28 days in a rat model^[^ ^71^ ^]^ as well as adhesion to stomach tissue for ≥ 10 days in a rabbit model.^[^ ^72^ ^]^ Taken together, the current findings illustrate the conceptual feasibility of sealant embedded electronic‐free diagnostic capabilities.

## 3 Conclusion

We present a dual modality gastric leak sensing concept that allows for unambiguous and timely identification of postsurgical anastomotic leaks, known as the most common fatal postsurgical complication in bariatric surgery. The presented sensors functionality under ultralow radiation dose CT and ultrasound allows for frequent patient screening, a key feature essential for rapid leak detection and optimal opportunity for timely treatment. Furthermore, both imaging modalities are fully established in the everyday clinical practice, considerably facilitating the clinical translation of the presented gastric leak sensors. The integration of these sensing elements into surgical sealants paves the road for leak detection prior to gastric fluid escape into the abdominal cavity, a phenomenon which otherwise greatly complicates treatment and carries substantial risks for the patient. We demonstrate patterning and geometric morphing of sensing elements under leak conditions for straightforward identification in medical images by the naked eye, as well as setting of the blueprint for artificial intelligence‐assisted recognition of impending leaks.

## 4 Experimental Section

### Chemicals and Materials

All materials were obtained from Sigma‐Aldrich (Merck) except for *N*‐acryloyl glycinamide (NAGA), which was obtained from Abmole (Belgium). *N*‐hydroxyl ethyl acrylate (NHEA) monomers were purified via filtration by basic alumina (Brockmann Grade I). All other products were used as obtained by the provider. Porcine tissue was obtained from a local slaughterhouse (Schlachtbetrieb Zürich, Switzerland). Preferably, tissues were used immediately after being obtained or after being thawed once. Simulated Gastric Fluid (SGF) was purchased from Biorelevant (UK) and prepared following the product instructions. Simulated Peritoneal Fluid was purchased from Biochemazone (Canada) and used as received.

### Hydrogel Preparation and Assembly

In a first step of fabricating the dual modality gastric leak‐detecting hydrogel patches, stock solutions of all constituent elements were prepared. This included a 20 wt% Acrylamide monomer (AAm) stock solution in milliQ water, a 1: 1 solution of purified *N*‐hydroxyl ethyl acrylate (NHEA) monomer in milliQ water, a 2 wt% *N*,*N*’‐Methylenebisacrylamide (mBAA) stock solution in milliQ water as a crosslinker, a UV photoinitiator stock solution prepared by dissolving 2‐Hydroxy‐4′‐(2‐hydroxyethoxy)‐2‐methylpropiophenone (Irgacure 2959) (4.825 mg, 21 µmol) in 1 mL of milliQ water, and finally a visible light photoinitiator stock solution prepared by dissolving 6.33 mg mL^−1^ of lithium phenyl‐2,4,6‐trimethylbenzoylphosphinate (LAP) in milliQ water. All solutions were stored at 0–4 °C. All photoinitiators were stored in the dark. In a second step, final polymerizable solutions were assembled using the prepared stock solutions. A polymerizable Aam solution was made by mixing 5 mL Aam stock solution, 108 µL mBAA crosslinker stock solution, and 500 µL UV photoinitiator (Irgacure 2959) stock solution. A polymerizable NHEA solution was made by mixing 4 mL NHEA stock solution, 53.32 µL mBAA 2 wt% crosslinker stock solution, and 302 µL UV photoinitiator (Irgacure 2959) stock solution. Finally, the mIPN polymerizable mix was assembled by combining 2 g NAGA, 4 mL milliQ water, and 450 µL of 6.33 mg mL^−1^ LAP. This placed all components to fabricate the dual modality gastric leak‐detecting hydrogel patches. The dual modality sensing elements were prepared by adding a 10 wt% dispersion of carbonate salt (barium or lanthanum) into the polymerizable Aam solution. The standard cylindrical sensing element geometry was prepared by adding 60 µL of the desired carbonate salt dispersion into a well of a 96‐Wellplate and polymerizing for 5 min under a UVASPOT 400/T mercury lamp at a distance of 30 cm from the light source. The spectrum of the lamp was controlled from 300 nm up to the visible by adding a H2 filter. Nonreactive reference elements were prepared by the same procedure, however, the 10 wt% carbonate salt component was replaced with a 5 wt% Tantalum (V) oxide component. The adhesive layer of the leak‐detecting hydrogel patch was prepared by adding 300 µL of the polymerizable AAm solution onto a Teflon mold of circular shape. If desired, a prepolymerized sensing element was placed centrically into the Teflon mold containing the polymerizable AAm solution and left to swell for 1 min. The adhesive layer was polymerized as previously described for 5 min, resulting in a fused adhesive‐sensing element layer. The nonadhesive backing was fabricated by following the same procedure. A total of 300 µL of the polymerizable NHEA solution was pipetted on top of the prefabricated adhesive‐sensing element layer and left to diffuse in the hydrogel layer for 1 min before being polymerized, as previously described. The prepared patches were covered with a polyethylene foil until their use. The mIPN polymerizable mix was assembled by combining 2 g NAGA, 4 mL milliQ water, and 450 µL of 6.33 mg mL^−1^ LAP. All prepared dual modality gastric leak‐detecting hydrogel patches were used immediately.

### Sensing Element Geometries

Standard cylindrical sensing elements were prepared with 10 wt% barium carbonate or lanthanum carbonate, as described above. The same procedure was performed with 5 wt% Tantalum(V) oxide as an unreactive reference. Ring‐shaped sensing elements with a reactive inner core were prepared by preparing standard cylindrical Tantalum (V) oxide elements. With the help of a 5 mm biopsy punch, the inner core of the element was removed and replaced with a carbonate salt (barium or lanthanum) solution and polymerized as previously described for 5 min. Ring‐shaped sensing elements with an unreactive inner core were prepared in the same manner by replacing the order of Tantalum(V) oxide and the carbonate salt (barium or lanthanum). Line‐shaped sensing elements were prepared in a custom‐built mold with dimensions 22 × 2 × 2 mm and subsequently cut to the desired length.

### Tissue Application of Hydrogel via mIPN Formation

All dual modality gastric leak‐detecting hydrogel patches were applied on porcine stomach serosa. First, the hydrogel patch was immersed in 1.5 mL of the mIPN polymerizable mix for a duration of 7 min. In addition, 150 µL (for a *d* = 2 circular hydrogel patch) of the mIPN solution, was dispersed dropwise onto the stomach serosa over the area of application. The hydrogel patch was placed onto the tissue, making sure that the adhesive layer had full contact with the stomach serosa. A quartz glass plate was used to keep the hydrogel patch in place and assure continuous firm contact of the hydrogel with the tissue. The dual modality sensing patches were irradiated for 5 min under unfiltered UV–visible light using a 2 × 6 W −365 nm VL‐206.BL lamp.

### Adhesion Measurements

The T peel test described in the ASTM standard F 2256‐05^[^ ^57^ ^]^ was used to measure the adhesion strength of hydrogel patches applied to porcine stomach tissue. Fresh porcine stomach tissue was cut into pieces of 10 × 5 cm using a scalpel. Hydrogel patches only consisting of the adhesive and the nonadhesive backing layers of dimensions 7 × 1.5 cm were prepared as described before. The hydrogel patches were applied to the stomach tissue as usually, all while keeping the final 2 cm of the hydrogel patches unattached to the tissue by placing polyethylene foil between the stomach tissue and the hydrogel patch prior to irradiation. To limit the deformation of the hydrogel patch and the tissue, rigid aluminum backings (10 × 5 × 0.2 cm) were superglued to the hydrogel patches and stomach tissue pieces, respectively. To perform the measurement, the nonadherent part of the hydrogel patch and the free tissue ends, including their respective alumina backings, were loaded into the devices mechanical grip clamps. During the experiment, standard force–strain curves were recorded while keeping the loading speed constant at 250 mm min^−1^. The ratio between the maximum standard force and the width of the sample were calculated to yield the T peel strength. More than three independent experiments were carried out to evaluate the T peel strength.

### H&E Stained Biopsy Slices—Hydrogel Patch and Tissue Interaction

A fresh porcine stomach was obtained from the slaughterhouse (Schlachtbetrieb Zürich, Switzerland) and used immediately. A circular (*d* = 2 cm) fully assembled gastric leak‐detecting hydrogel patch containing a barium carbonate dual modality sensing element was attached to the tissue as described above. After successful application, biopsy samples of the tissue adhered hydrogel were obtained with an 8 mm biopsy punch. Samples were places in 4% formalin solution. After 24 h, any excess swollen hydrogel was removed from the sample using a scalpel, while making sure that the hydrogel‐tissue interface was kept intact. The samples were continuously stored in 4% formalin solution. All samples were sent to Sophistolab AG, Muttenz, Switzerland, where they were placed in paraffin block, cut and stained using H&E. Imaging of the biopsy slices was performed in‐house.

### Cell Handling and Cytotoxicity Experiments

The fully supplemented cell growth medium for the normal human dermal fibroblast cell line (NHDF, PromoCell C‐12302) consisted of Dulbecco's Modified Eagle's Medium (high glucose, Sigma‐Aldrich) supplemented with 10% fetal calf serum (Sigma‐Aldrich), as well as 1% L‐glutamine (Sigma‐Aldrich) and penicillin‐streptomycin (Sigma‐Aldrich). Cells were kept at 37 °C under a humidified atmosphere, containing 5% CO_2_ and were subcultured at 70–80% confluency by treatment with 0.5% Trypsin‐EDTA (Sigma‐Aldrich). To assess the cytotoxicity and viability after metal salt treatment, 10 000 NHDF cells in 100 µL were seeded in black 96 well plates with a transparent bottom. After 24 h attachment time, the cell medium was replaced, and cells were treated for 24 h with different salt concentrations (0, 0.125, 0.25, 0.5, 1, 2 mm) of BaCl_2_(H_2_O)_2_ (*M*_w_ = 244.26 g mol^−1^) and LaCl_3_(H_2_O)_7_ (*M*_w_ = 371.37 g mol^−1^) dispersed in cell medium with a total of 10% MilliQ water per treatment concentration. For cytotoxicity evaluations, 50 µL of supernatant from each experimental well was transferred to a transparent 96 well plate and incubated with 50 µL lactate dehydrogenase substrate (LDH, CytoTox 96 Non‐Radioactive Cytotoxicity Assay, Promega, G1780) in the dark for 30 min at room temperature. Thereafter, the absorbance at 490 nm was measured using a Mithras LB 943 Multimode Plate Reader. LDH release was calculated by subtracting the mean background values from the absorbance values and normalizing the results to the positive control, which were Triton X‐100 treated (0.1%), lysed cells. For the cell viability assessment, the rest of the cell medium per experimental well was replaced by 50 µL fresh cell medium and 50 µL CellTiter‐Glo reagent (Promega G7571). The luminescence was measured using the plate reader after 10 min shaking in the dark. The luminescence was then normalized to the luminescence of 100% viable control cells.

### Ultrasound and CT Measurements

Ultrasound phantoms (12 × 6 × 6 cm) were made from 20 wt% AAm. Phantoms were produced with either cylindrical cavities (3 × 2.5 cm) or rectangular cavities (6 × 5 × 3 cm), which were used as sample holders. The sample holders were filled with distilled water during measurements. The ultrasound image was acquired with a Clarius C3 HD_3_ Multipurpose Scanner (purchased from Meditron SA. Morges Switzerland). To guarantee ultrasound image comparability, the ultrasound gain was manually set to 52%, and the image zoom was kept at 7.8 cm. Ultrasound data were recorded by capturing images from the ultrasound B‐mod feed and used as provided by Clarius Cloud. The ultrasound signal was quantified by converting the recorded images to 8‐bit greyscale images and computing the mean pixel value over a manually selected area of the image. This was done with an in‐house developed python tool. Quantitative ultrasound results were obtained by considering at least three independent experiments.

Samples were placed in well plates for CT measurements and scanned using a third‐generation energy integrating detector CT (Somatom Edge Plus, Siemens Healthcare GmbH, Forchheim, Germany). Scans were acquired using a tube voltage of 120 kV and a fixed tube current of 500 mAs. Anthropomorphic phantom scans with a 3D printed insert containing samples were acquired with a tub voltage of 120 kV and a reference tube current of the automated tube current modulation system (CAREDose4D, Siemens) to achieve a volume CT dose index (CTDI_vol_) of 10 mGv. A CTDI_vol_ of 10 mGy is considered the reference dose level for abdominal CT.^[^ ^63^ ^]^ In addition, anthropomorphic phantom scans were acquired with a fixed tube voltage of 120 kV and with the minimal tube current allowed by the scanner, i.e., 6 mAs, resulting in an ultralow dose scan with a CTDI_vol_ of 0.42 mGy. All images were reconstructed with a slice thickness of 0.5 mm and an increment of 0.5 mm using the quantitative Qr66 kernel and advanced iterative reconstruction (ADMIRE) at a strength level of 3. Average attenuation values (in HU) including the entire sensing element volume were measured with OsiriX MD (Pixmeo, Switzerland).^[^ ^73^ ^]^ All quantitative CT results were obtained by considering at least three independent experiments.

### Gastric Leak Sealing Model

Pieces of freshly thawed porcine stomach tissue were cut into 4 × 4 cm pieces. For ease of handling, the inner mucosal layer of the stomach was removed. A defect centrally located on the tissue piece was created with an 8 mm biopsy punch. Circular dual modality gastric leak‐detecting sealant hydrogel patches were assembled as previously described along with a standard cylindrical barium carbonate sensing element placed centrically into the adhesive layer of the hydrogel patches. The perforation was then sealed by a hydrogel patch, all while ensuring that no excess mIPN solution occluded the tissue defect and guaranteeing that the sensing element was located directly over the perforation. The hydrogel sealed tissue piece was placed into an in‐house designed tissue holder allowing to bring the mucosal side of the tissue piece in contact with a 20 mL fluid reservoir. The tissue holder was wrapped in polyethylene foil to avoid drying of the stomach tissue and/or hydrogel patch. For the gastric leak model, the fluid reservoir was filled with SGF. For the control model, the fluid reservoir was filled with PBS. The tissue holders were mounted onto an orbital shaker at 20 rpm at 37 °C overnight. The gastric leak detecting sensing elements were subsequently evaluated under ultrasound and CT imaging following the aforementioned methodology.

### In Vivo Study in a Piglet Model

An in vivo proof of principle in a piglet model was performed in collaboration with the Biomedical Center, Faculty of Medicine in Pilsen, Czech Republic. The study was certified by the Commission of Work with Experimental Animals at the Medical Faculty of Pilsen, Charles university (project ID: MSMT‐15629/2020‐4), and under control of the Ministry of Agriculture of the Czech Republic. All procedures were performed in compliance with the law of the Czech Republic, which is compatible with the legislation of the European Union. Proof‐of‐concept experiments were carried out in two pigs and data for one representative pig are shown. Healthy male Prestice black‐pied pigs (14 weeks old, weighing 30–40 kg) were premedicated using ketamine (Narkamon 100 mg mL^−1^, BioVeta a.s. Ivanovice na Hané, Czech Republic) and azaperone (Stresnil 40 mg mL^−1^, Elanco AH, Prague, Czech Republic) administered intramuscularly. General anesthesia was introduced and maintained by propofolum MCT/LCT (Propofol 2% MCT/LCT Fresenius Medical Care a.s.). Nalbuphin (Nalbuphin, Torrex Chiesi CZ s.r.o., Prague, Czech Republic) was used for analgesia. Orogastric tube was used prior to surgery to eliminate present gastric content. In order to simulate a gastric leak, the stomach of the piglet was filled with 1.5 L of simulated gastric fluid (SGF). Samples of the fluid were taken each 30 min to monitor the acidity changes of the fluid. Laboratory pH meter (Jenway model 550 pH meter, Cole‐Parmer Instrument Company LTD, UK) was used to evaluate the samples. Midline incision laparotomy was performed to enter the abdominal cavity. Small pockets on the stomach were created by seromuscular incision sparing the mucosa. A smaller 1 cm defect in the mucosa was created afterward for patch implantation. Circular dual modality gastric leak detecting hydrogels containing a ring‐shaped sensing element with a 10 wt% barium carbonate core and a 5 wt% tantalum oxide outer ring, preinfused with mIPN and polymerized, were applied to the tissue pockets over the mucosal defect via glyconate monofilament 4/0 suture (Monocryl 4/0, B. Braun Medical s.r.o., Prague, Czech Republic). After successfully securing the hydrogel patches in place, the tissue pockets were closed using the same type suture line. The abdominal wall was closed by polydioxanone monofilament 1 suture (PDS II, 1, Ethicon, Johnson & Johnson, NJ). A first CT scan (Siemens Healthcare) of the piglet was performed directly after applying the dual modality gastric leak sensing hydrogels. The CT acquisition parameters were set to a 70 kV tube voltage and a 250 mAs fixed tube current. Additional CT scans were performed at hours 3 and 6 post SGF administration using the same protocol as stated above. Additionally at hour 6 post SGF administration, the stomach was investigated via ultrasound using the Clarius L7 HD_3_ scanner (purchased from Meditron SA. Morges, Switzerland) through the abdominal wall, locating the activated dual modality gastric leak sensors. At this point, the animal was reopened via the same incision, all gastric pockets were opened, and all dual modality gastric leak detecting hydrogels were surgically removed for visual inspection. The animal was then immediately sacrificed using a cardioplegic solution and tissue samples were harvested. The entirety of the experiment was documented via photography.

### Statistical Analysis

No data preprocessing was performed. Data were used for analysis as recorded. Analyzed data are shown as mean ± SD, except data in Figure S8 (Supporting Information) where the variable uncertainties Δ*x_i_* (given by the standard deviation of independent measurements) were propagated, yielding $\Delta f^{2}\;=\;\sum_{i}{\left(\frac{\partial f}{\partial x_{i}}\Delta x_{i}\right)}^{2}$ along with analyzed data shown as *f*(x) ± Δ*f*. Sample sizes are indicated in the figure captions. Data were analyzed by using IPython, Numpy, SciPy, OpenCV, and Matplotlib.

## Conflict of Interest

B.S., A.H.C.A., and I.K.H. declare that patent applications have been filed covering all parts of the adhesion and sensing technology reported in this publication. The patents have been filed by ETH Zurich and Empa (Alexandre H.C. Anthis, Martin T. Matter and Inge K. Herrmann, PCT/EP2022/051137, patent field and Alexandre H.C. Anthis, Benjamin Suter and Inge K. Herrmann, PCT/EP2022/051141, patent filed). All other authors report no conflict of interest.

## Supporting information

Supporting Information

## Data Availability

The data that support the findings of this study are available in the supplementary material of this article.
