# Supplementary material for: Surgical Sealant with Integrated Shape‐Morphing Dual Modality Ultrasound and Computed Tomography Sensors for Gastric Leak Detection
Source: Adv Sci (Weinh). 2023 Jun 5;10(23):2301207. doi: 10.1002/advs.202301207 (PMC10427398; doi:10.1002/advs.202301207)
Supplement: Supplementary file 1 — Supporting Information [file ADVS-10-2301207-s001.pdf]

ADVANCED SCIENCE
Open Access

Supporting Information

for *Adv. Sci.*, DOI 10.1002/advs.202301207

Surgical Sealant with Integrated Shape-Morphing Dual Modality Ultrasound and Computed Tomography Sensors for Gastric Leak Detection

Benjamin Suter, Alexandre H. C. Anthis, Anna-Katharina Zehnder, Victor Mergen, Jachym Rosendorf, Lukas R. H. Gerken, Andrea A. Schlegel, Eva Korcakova, Vaclav Liska and Inge K. Herrmann**

Supporting Information

Surgical Sealant with Integrated Shape-Morphing Dual Modality Ultrasound and Computed Tomography Sensors for Gastric Leak Detection

Benjamin Suter,[1,2] Alexandre H.C. Anthis, [1,2,] Anna-Katharina Zehnder, [1] Victor Mergen, [3] Jachym Rosendorf, [4,5] Lukas R.H. Gerken, [1,2] Andrea A. Schlegel,[6,7] Eva Korcakova, [4,5] Vaclav Liska, [4,5] Inge K. Herrmann[1,2,]* *

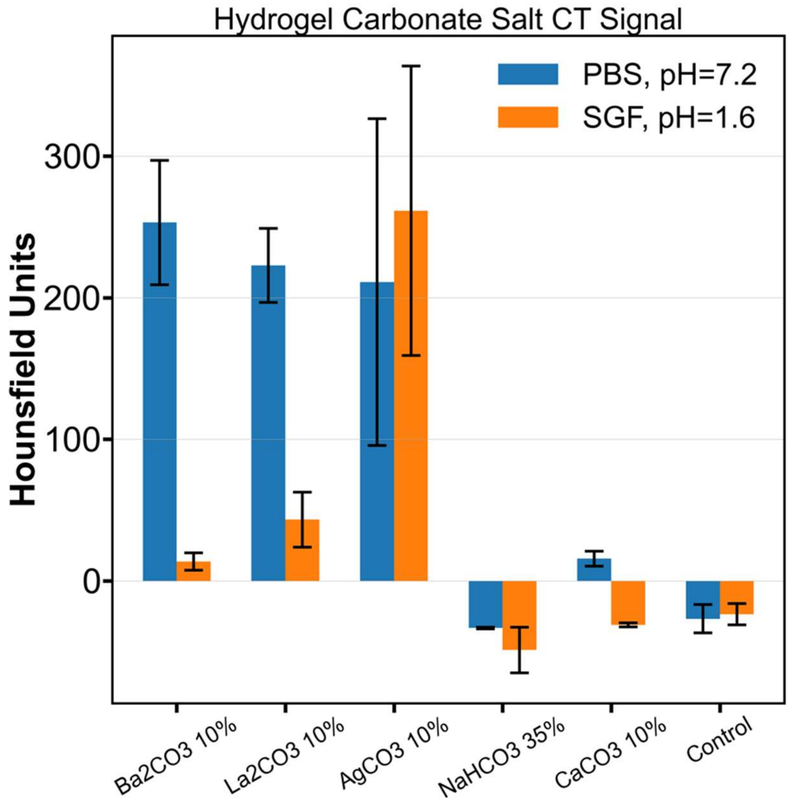

Figure S1: Carbonate screening. *We investigated the change in computed tomography (CT) attenuation of different carbonate salts embedded in a hydrogel matrix after being incubated overnight in phosphate-buffered saline (PBS) and simulated gastric fluid (SGF) respectively. All concentrations are given in wt.% of the final 150 µL Poly-acrylamide-kappa-carrageenan (20 wt.% AAm, 1 wt.% K). Measurements were taken with n=2 where both samples were incubated in the same 10 mL volume of fluid. N=3 independent experiments performed per condition.*

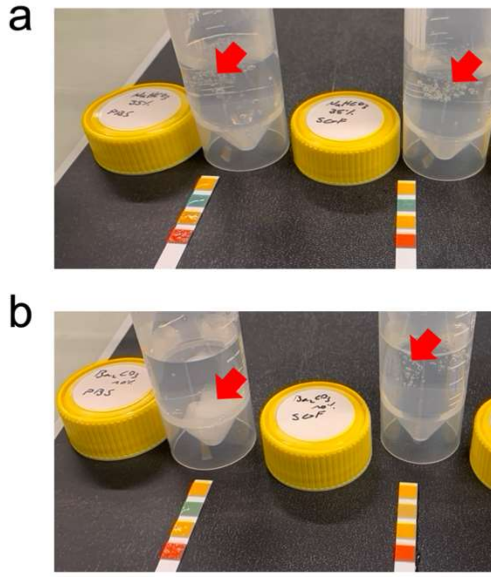

Figure S2: Reaction under no-leak conditions of $NaHCO_3$. *(a) Left to right: phosphate-buffered saline (PBS) and simulated gastric fluid (SGF). In both conditions the sodium bicarbonate ($NaHCO_3$) containing hydrogel can be seen floating at the top of the fluid with carbon dioxide (CO_2) bubbles clearly contained in the hydrogel. This is an unwanted effect. For robust ultrasound detectability of gastric leaks, we only want CO_2 bubble formation under contact with SGF. (b) 10 wt.% barium carbonate ($BaCO_3$) containing hydrogel incubated in PBS (left) and SGF (right). Here, only the sample incubated in SGF can be seen floating at the top of the fluid with CO_2 bubbles encapsulated in the hydrogel.* Representative images from N=3 samples shown.

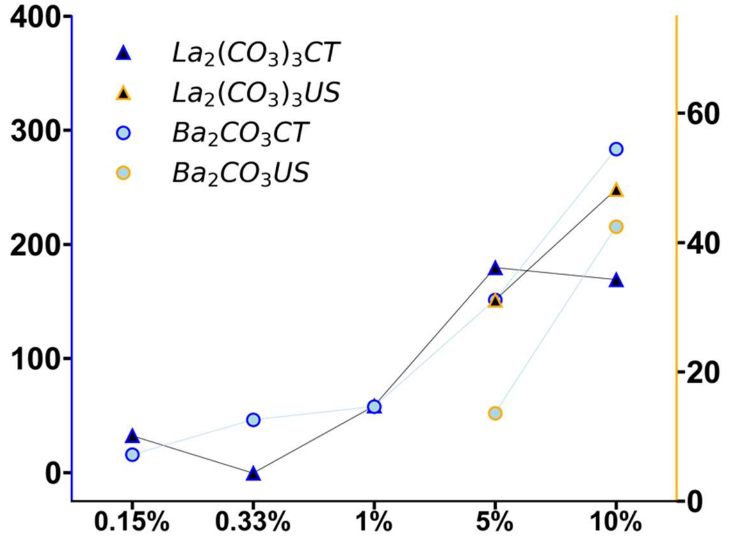

Figure S3: CT and ultrasound contrast dependance on carbonate salt weight concentration. *Absolute value of the signal change between samples incubated in phosphate-buffered saline (PBS) and samples incubated in simulated gastric fluid (SGF) under computed tomography (CT) and ultrasound (US). At 10wt.% satisfactory changes in contrast were achieved in both imaging modalities. For barium carbonate ($BaCO_3$) as well as for lanthanum carbonate (La_2CO_3). N=3 independent experiments performed per condition. Data are represented by their mean value.*

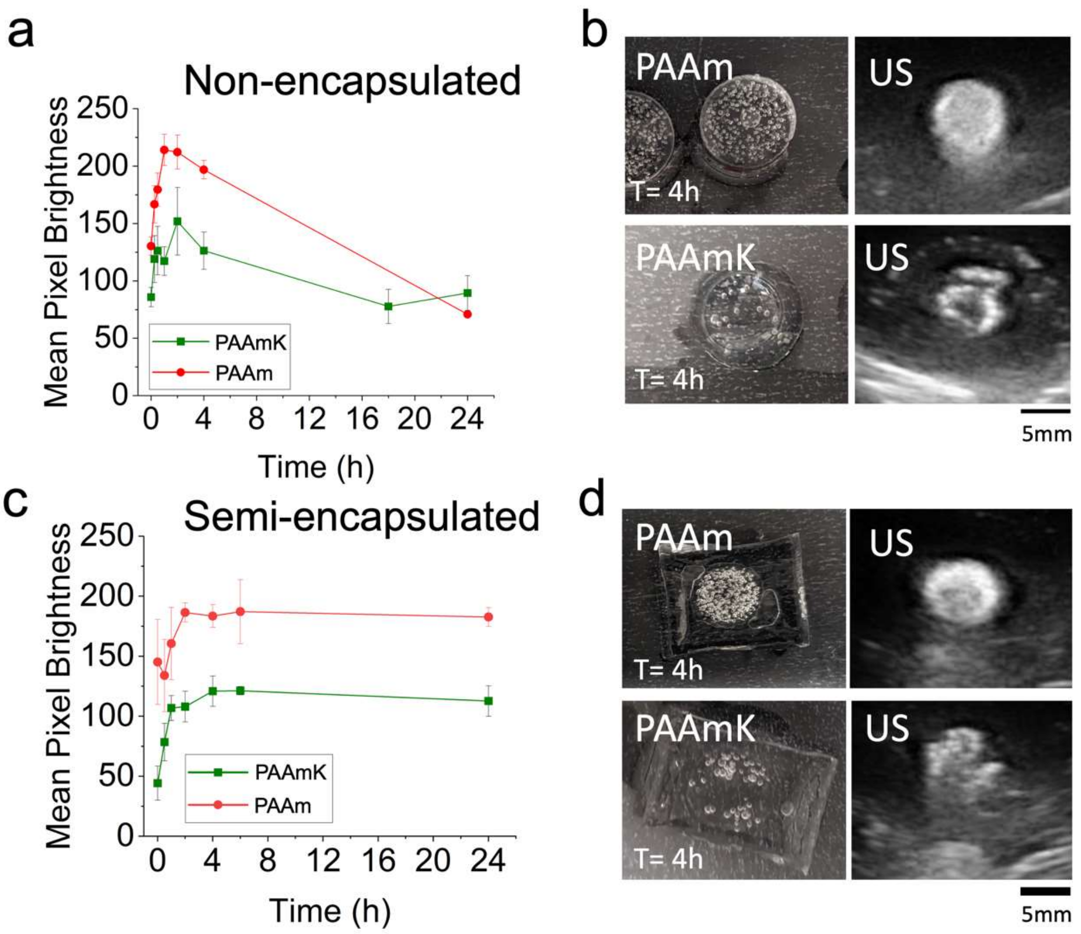

Figure S4: Sensing element matrix choice. *60 µL hydrogels containing 10wt.% barium carbonate ($BaCO_3$). (A) Time resolved ultrasound contrast when incubated in simulated gastric fluid (SGF). Poly-acrylamide (PAAm) matrix shows a much higher contrast peak when compared to a Poly-acrylamide-Kappa-carrageenan (PAAmK) matrix. Both elements lose their contrast over a span of 24 h. (B) Visual and ultrasound comparison of the two matrices. PAAm shows a higher number of entrapped carbon dioxide (CO_2) bubbles as well as a higher homogeneity resulting in a better identifiable ultrasound (US) image. (C) Same elements as in (A) but now semi encapsulated in a secondary PAAm matrix. Both elements now hold a steady contrast over 24 h after full reaction. (D) Visual and ultrasound image of the two semi encapsulated elements. Again, the pure PAAm matrix yields a larger number of encapsulated bubbles and a more homogeneous distribution of the bubbles, providing a better ultrasound image. N=3 independent experiments performed per condition.*

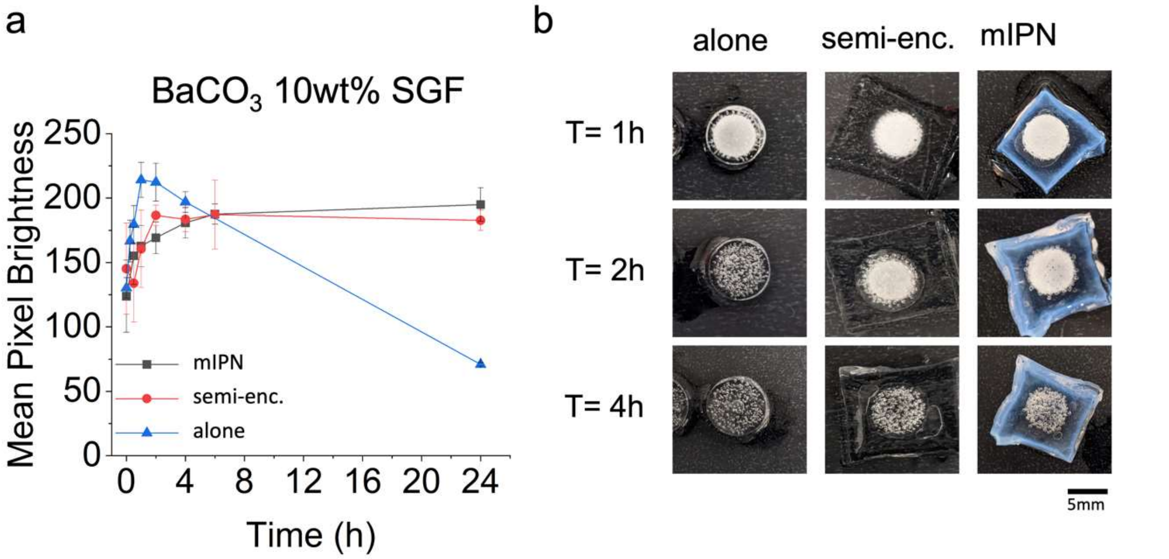

Figure S5: Sensing element encapsulation. *Comparison of 60 µL hydrogels containing 10wt.% barium carbonate ($BaCO_3$) alone, semi encapsulated in a poly-acrylamide (PAAm) matrix and semi encapsulated in a PAAm matrix along with the polymerized mutually interpenetrating network (mIPN) under a time resolved ultrasound signal when incubated in simulated gastric fluid (SGF). (A) The kinetics of the semi encapsulated elements (with and without mIPN) are nearly identical. This lets us conclude that the mIPN does not obstruct the reactivity of the sensing elements. Furthermore, the increase in signal stability due to semi encapsulation is shown. (B) Visual image of the three different hydrogel samples. N=3 independent experiments performed per condition.*

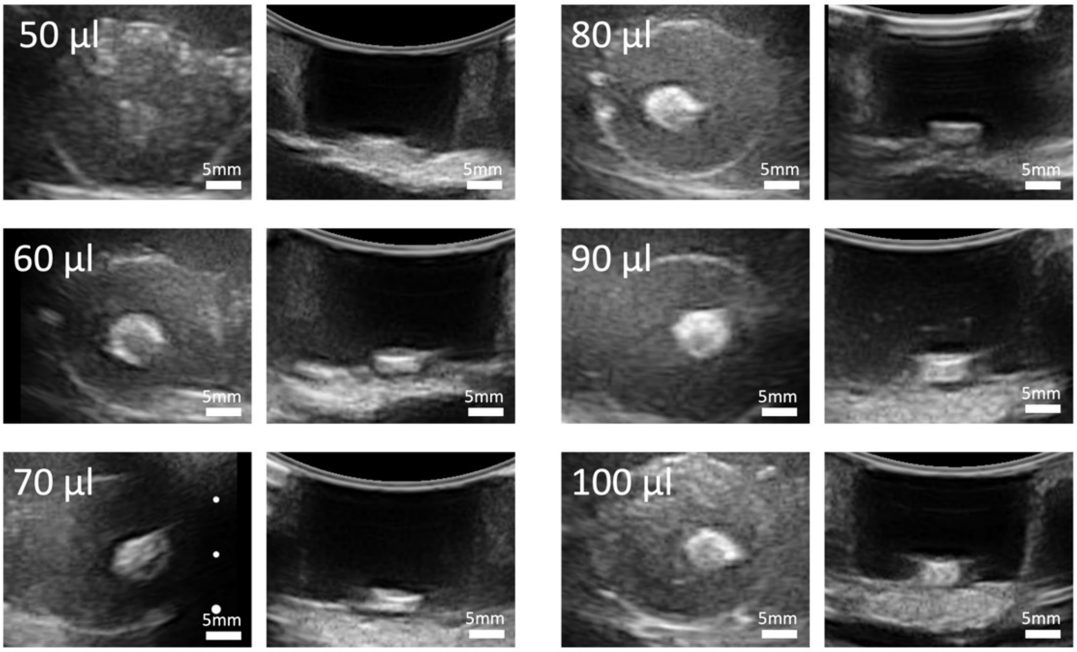

Figure S6: Sensing element volume optimization for maximal visibility under clinical ultrasound. *Different volumetrically sized sensing elements containing 10 wt.% barium carbonate ($BaCO_3$) evaluated under ultrasound for visibility in their unreacted form. 60 µL hydrogels were chosen to be the smallest hydrogels still visible under ultrasound. Representative images from N=3 samples shown..*

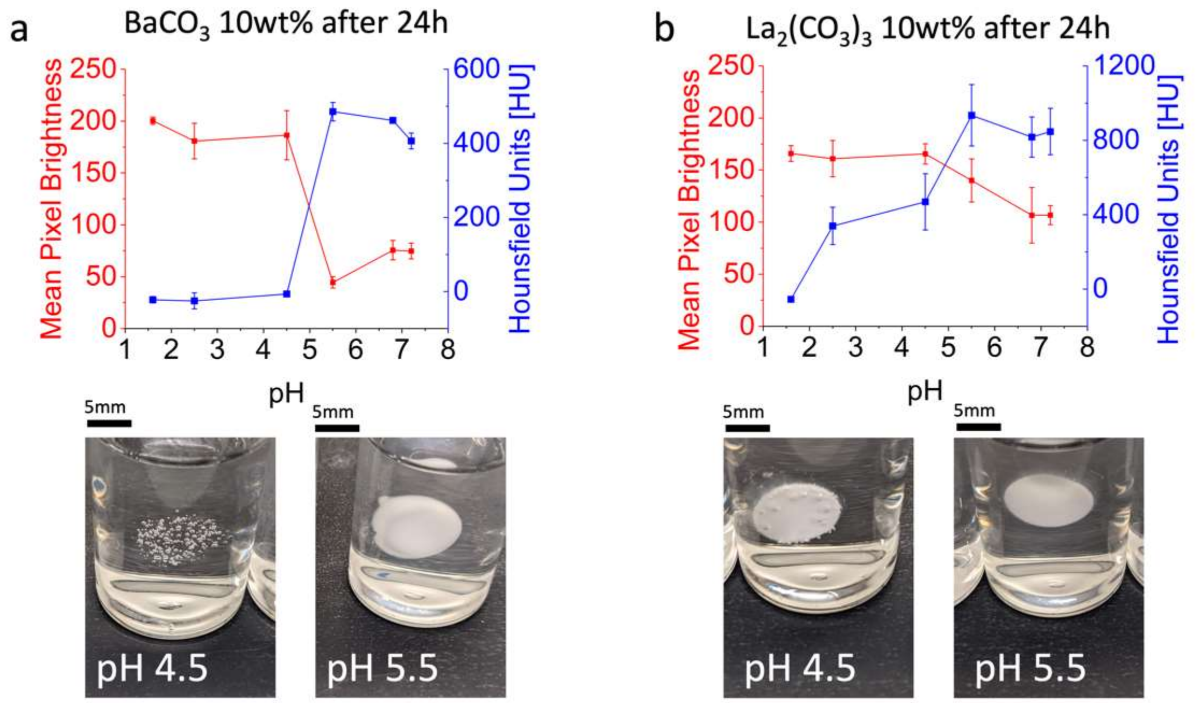

Figure S7: pH response of $BaCO_3$ and $La_2(CO_3)_3$ along with visual example of sensing element. *60 µL poly-acrylamide (PAAm) hydrogels with 10 wt.% of barium carbonate ($BaCO_3$) and lanthanum carbonate ($La_2(CO_3)_3$) respectively were incubated in pH buffers from pH 1.6 (simulated gastric fluid) up to pH 7.2 (phosphate-buffered saline). $BaCO_3$ shows reactivity over a broad range of pH values as well as a sharp cutoff. $La_2(CO_3)_3$ shows a much flatter pH dependent contrast curve, making it less suited for leak sensing. N=3 independent experiments performed per condition.*

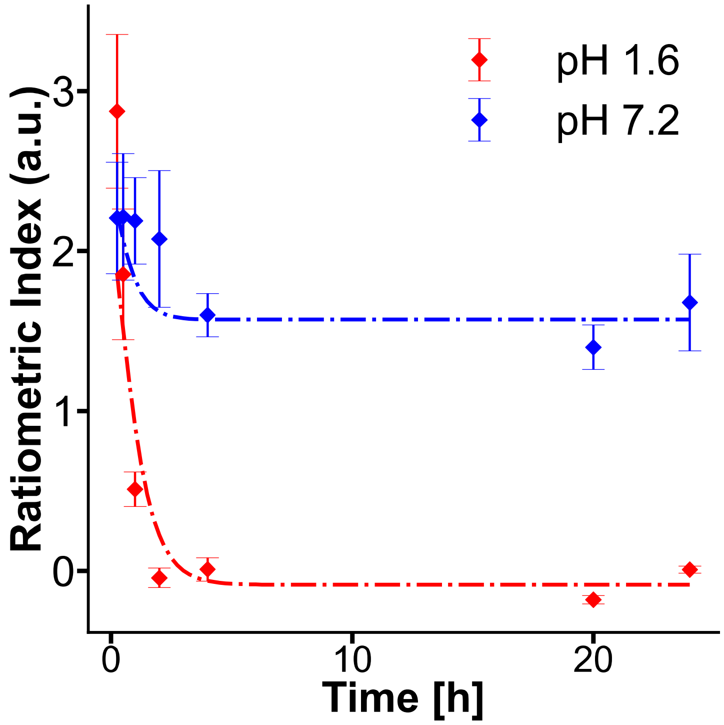

Figure S8: Ratiometric CT attenuation evolution for 10 wt.% $BaCO_3$. *The computed tomography (CT) signal of a 10 wt.% barium carbonate ($BaCO_3$) sensing element was normalized with the non-reactive CT signal of a 5 wt.% tantalum oxide (Ta_2O_5) element. This was done with the intention of reducing the signal decrease cause by swelling of the hydrogel matrix. In order to fully remove the signal contribution caused by swelling. N=3 independent experiments performed per condition.*
